# Supplementary figures and images for: HIV-1 Vpr suppresses expression of the thiazide-sensitive sodium chloride co-transporter in the distal convoluted tubule
Source: PLoS One. 2022 Sep 21;17(9):e0273313. doi: 10.1371/journal.pone.0273313 (PMC9491550; doi:10.1371/journal.pone.0273313)

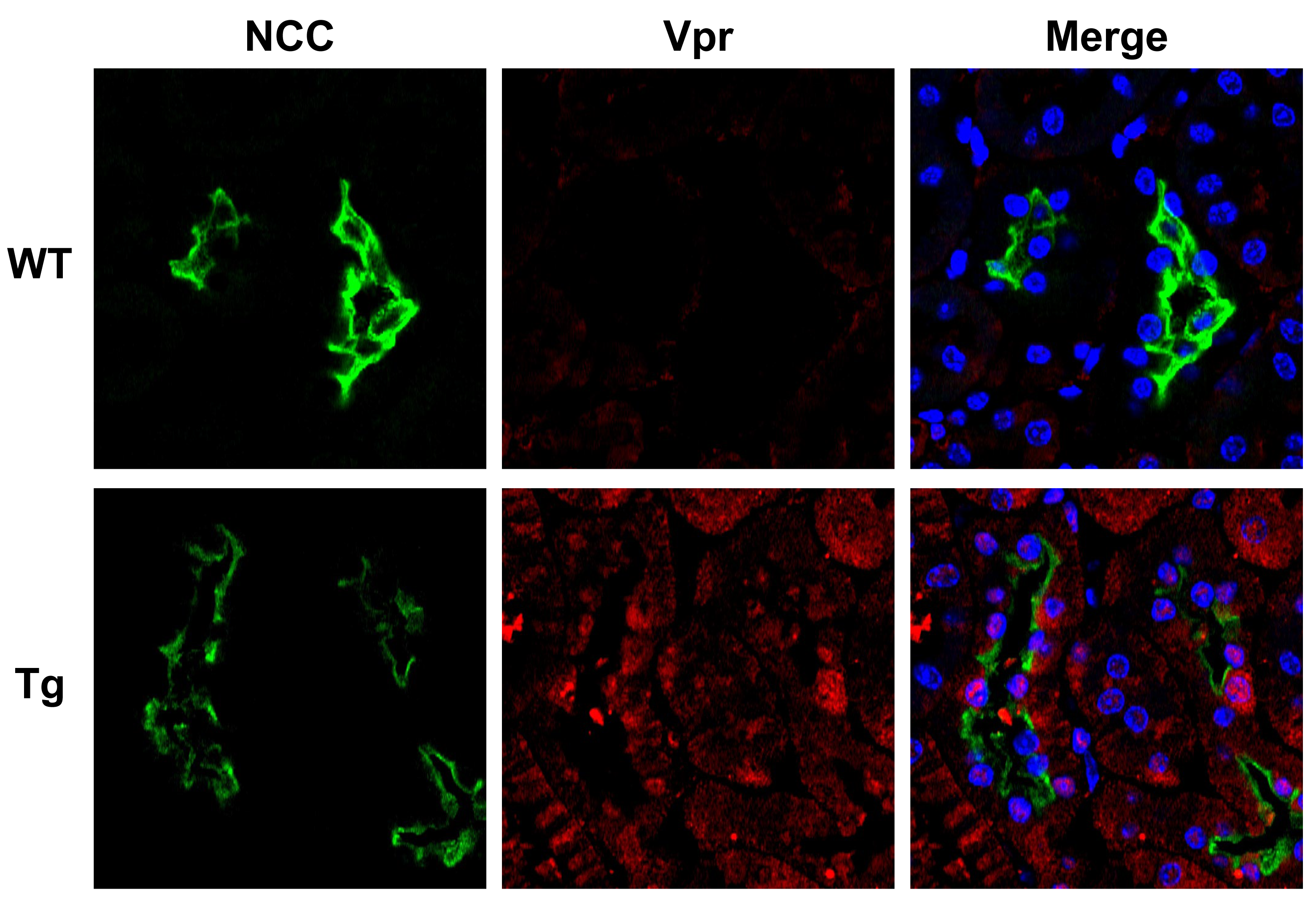

Supplement: S1 Fig — Mouse kidney sections were stained using NCC (green) or Vpr (red). NCC is expressed in the apical membrane of distal convoluted tubule in both WT and Tg mice, whereas Vpr is expressed in tubules in Vpr Tg mice but not in WT mice. Nuclei were stained in blue with Hoechst 33342. Magnification x300. (TIF) [file pone.0273313.s001.tif]

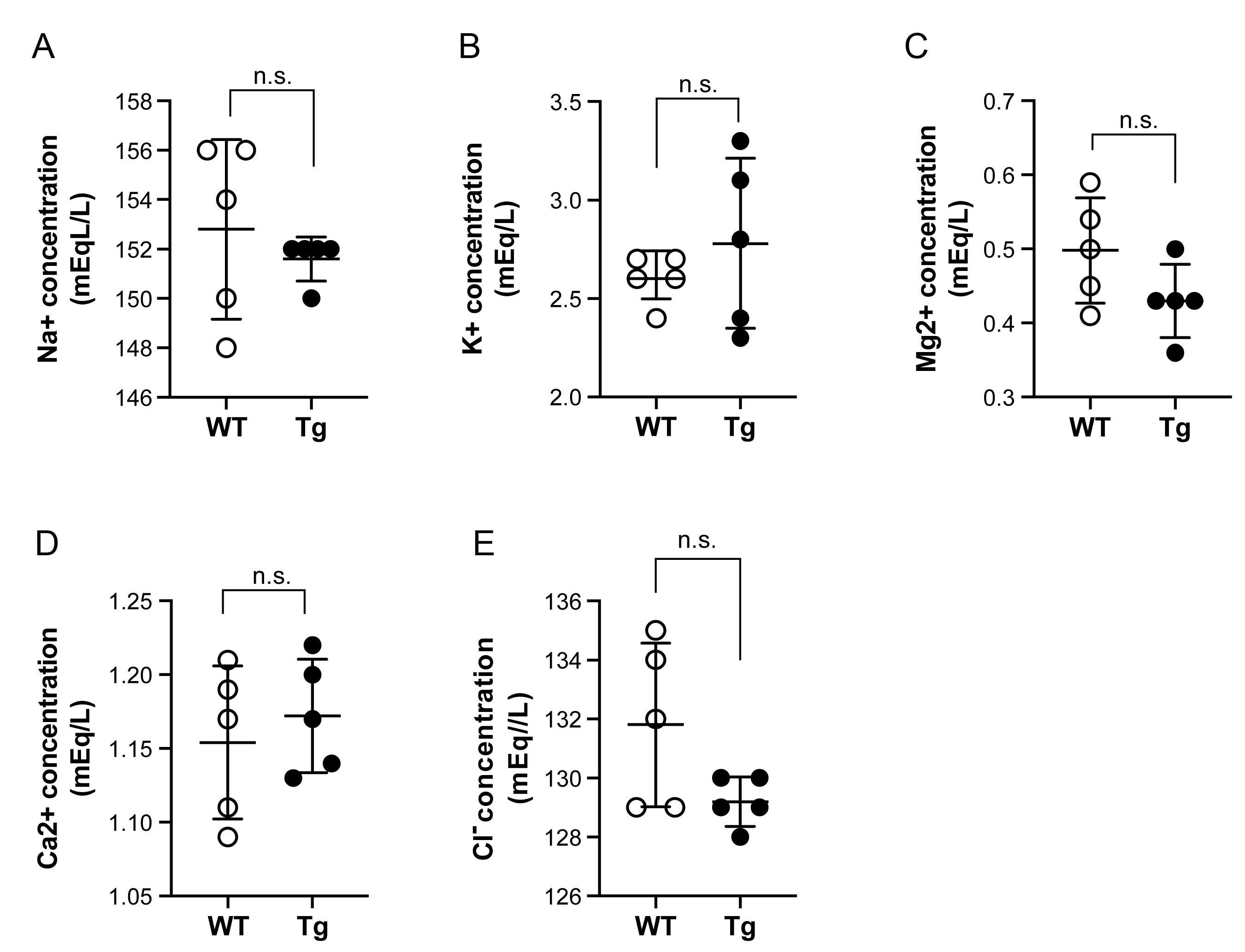

Supplement: S2 Fig — (A) Na+, (B) K+, (C) Mg2+, (D) Ca2+, and (E) Cl- concentration had no difference between Tg and WT mice. n = 5. n.s., not significant, Student’s t test. The electrolytes were measured at NIH Clinical Center Clinical Laboratory (Bethesda, MD). (TIF) [file pone.0273313.s002.tif]

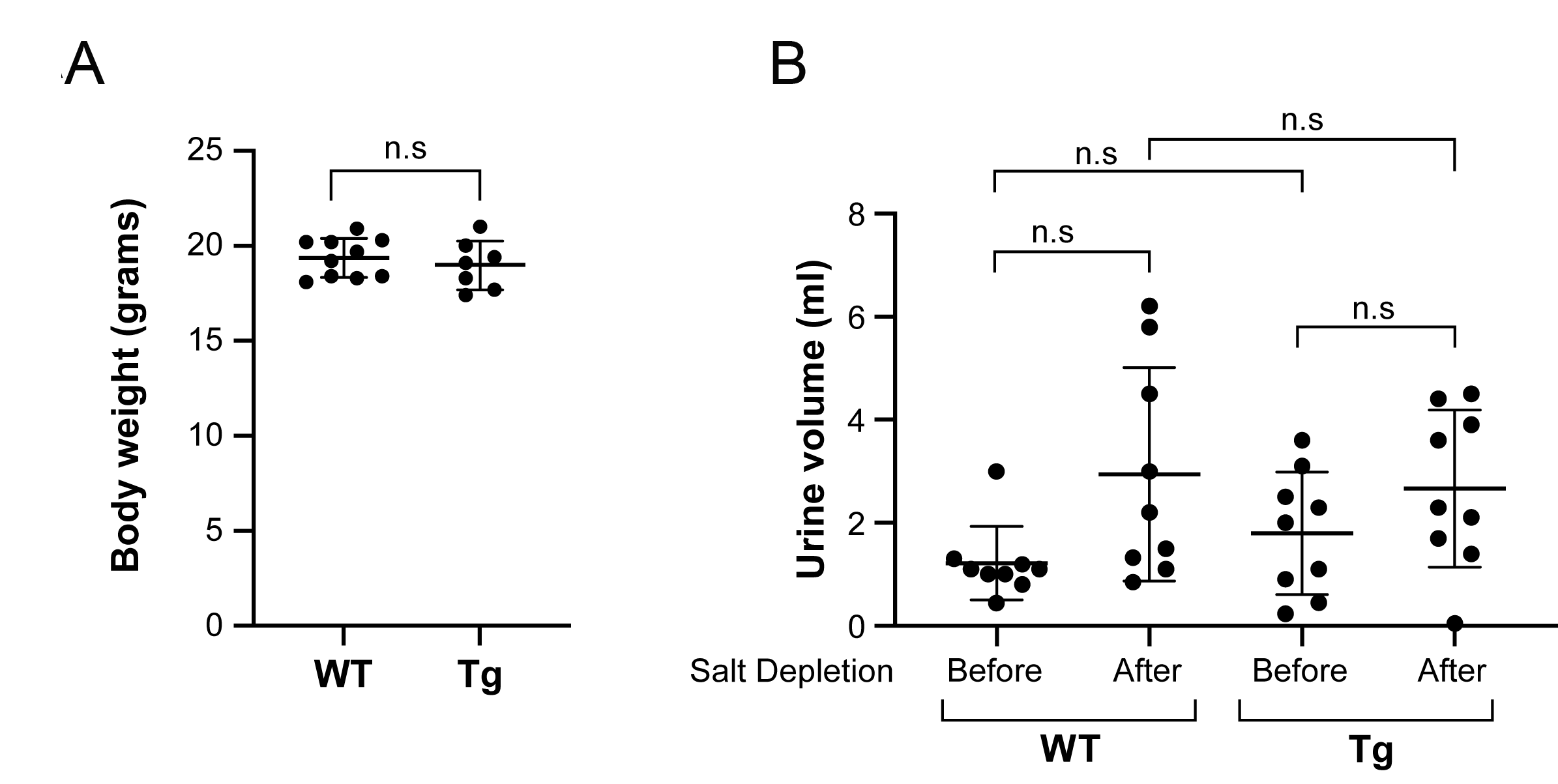

Supplement: S3 Fig — (A) Four days after the low salt diet the mice were weighed. n = 7–10. n.s., not significant, Student’s t test. (B) Mouse were placed in metabolic cages and overnight urine was collected before and after the mice were placed on low salt diet. n = 9. n.s., not significant, ANOVA, Bonferroni correction. (TIF) [file pone.0273313.s003.tif]

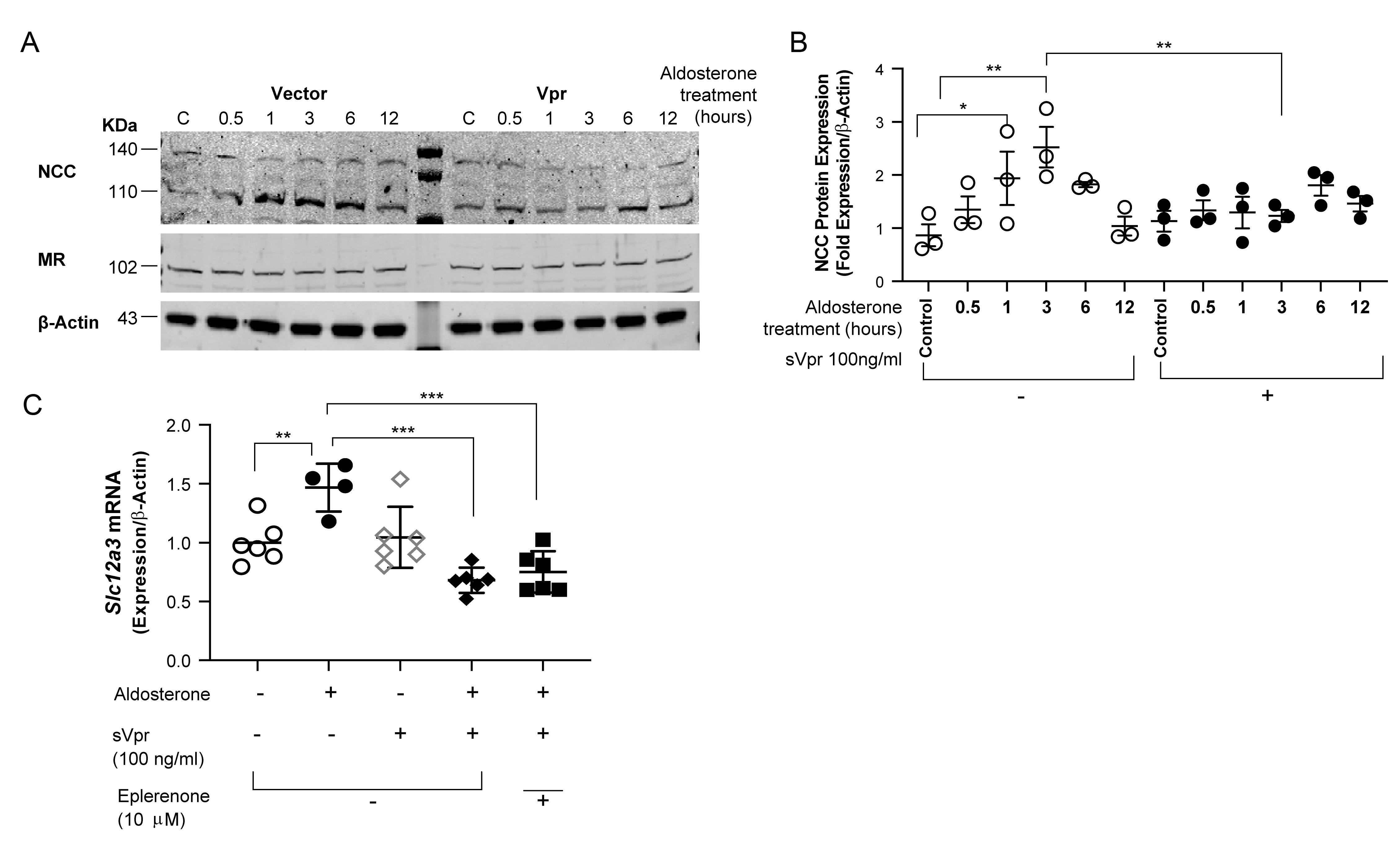

Supplement: S4 Fig — (A) Human DCT cells were treated with or without soluble Vpr (sVpr, 100ng/ml) for 24-hr followed by aldosterone treatment for the time indicated. Protein expression of NCC and MR were determined by immunoblotting. (B) The density of NCC was quantified by densitometry. The aldosterone-enhanced NCC protein expression, with a peak at 3-hr treatment, was abolished by sVpr (100ng/ml). n = 3. *P < 0.05, **P <0.01. (C) Human DCT cells were treated with sVpr, aldosterone, or eplerenone (10μM), individually or in combination as indicated. Expression of SLC12A3 mRNA was determined by qRT-PCR and normalized to β-actin mRNA. The aldosterone-induced increase in SLC12A3 mRNA expression was attenuated by Vpr, but eplerenone had no further effect on SLC12A3 mRNA expression. n = 4–6. **P <0.01, ***P < 0.001, ANOVA, Bonferroni correction. (TIF) [file pone.0273313.s004.tif]

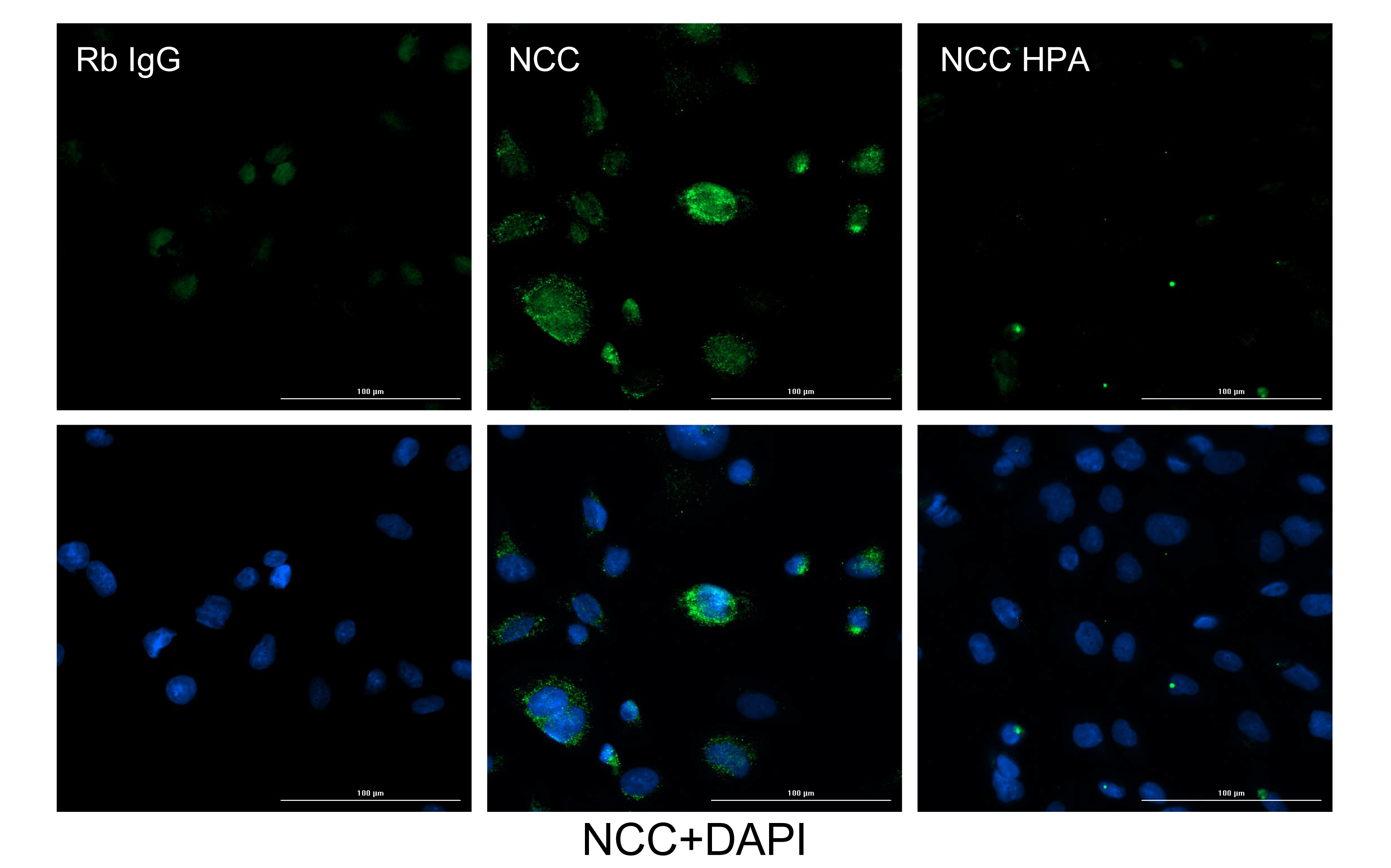

Supplement: S5 Fig — The immortalized human renal distal convoluted tubule cells were previously characterized [8]. Here, additional immunofluorescence staining was performed. Green, anti-NCC antibody (Cat. No.: AB3553, 1:500 dilution); Blue, nucleus. Scale bar, 100 μm. (TIF) [file pone.0273313.s005.tif]
