# Supplementary material for: HIV-1 Vpr suppresses expression of the thiazide-sensitive sodium chloride co-transporter in the distal convoluted tubule
Source: PLoS One. 2022 Sep 21;17(9):e0273313. doi: 10.1371/journal.pone.0273313 (PMC9491550; doi:10.1371/journal.pone.0273313)
Supplement: S3 Table — (DOCX) [file pone.0273313.s008.docx]

| Antibody | Source | Cat. No. | Working condition | Note |
| --- | --- | --- | --- | --- |
| Vpr (9-F12) | Dr. Kopp’s laboratory & Maine Biotechnology | Not available | 1:200 | Mouse monoclonal |
| Anti-NCC | Dr. Knepper's laboratory | 4735 FR6 | 1:1000 | Rabbit Polyclonal |
| Anti -NCC (for staining hDCT cells)* | Millipore | AB3553 | 1:500 | Rabbit Polyclonal |
| Anti-MR (MCR) | Santa Cruz Biotechnology | sc-11412  (H-300) | 1:1000 | Rabbit Polyclonal |
| Anti-FLAG | Sigma | F1804 | 1:2000 | Mouse monoclonal |
| Anti-Na^+^/K^+^-ATPase | Millipore | 06-520 | 1:1000 | Rabbit Polyclonal |
| Anti-β-actin | Santa Cruz Biotechnology | Sc-47778 | 1:2000 | Monoclonal mouse |

* This antibody was used for immunostaining of the human DCT cells.
