# Supplementary material for: HIV-1 Vpr suppresses expression of the thiazide-sensitive sodium chloride co-transporter in the distal convoluted tubule
Source: PLoS One. 2022 Sep 21;17(9):e0273313. doi: 10.1371/journal.pone.0273313 (PMC9491550; doi:10.1371/journal.pone.0273313)
Supplement: S4 Table — (DOCX) [file pone.0273313.s009.docx]

| **Target gene** | **Primer names** | **Primer sequences** |
| --- | --- | --- |
| *Actb*  (mus musculus) | mβ-actin-fwrd  mβ-actin-revs | 5’-CCACCATGTACCCAGGCATT -3’  5’-AGGGTGTAAAACGCAGCTCA-3’ |
| *Slc12a3*  (mus musculus) | mSlc12a3_fwrd  mSlc12a3_revs | 5’- TACATGCGCACCTTCGGTTA -3’  5’- GGTGGCTACCTTCCTGCTTG -3’ |
| *ACTB*  (homo sapiens) | hβ-actin _fwrd  hβ-actin _revs | 5'- GAGCACAGAGCCTCGCCTTT -3’  5'- AGAGGCGTACAGGGATAGCA -3’ |
| *SLC12A3*  (homo sapiens) | hSlc12a3_fwrd  hSlc12a3_revs | 5'- CACTGGCTGACCTGCACTC -3’  5'- ACCAGCCCATCAGTCATCTC -3’ |
