# Supplementary material for: HIV-1 Vpr suppresses expression of the thiazide-sensitive sodium chloride co-transporter in the distal convoluted tubule
Source: PLoS One. 2022 Sep 21;17(9):e0273313. doi: 10.1371/journal.pone.0273313 (PMC9491550; doi:10.1371/journal.pone.0273313)
Supplement: Supplementary file 4 [file pone.0273313.s006.docx]

Matrix	Detailed Matrix Information	Tissue	Opt.	tart positic	nd positio	chor positi	Strand	Core sim.	Matrix sim.	at. sim. - o	Evidence #	Evidence	Sequence	Primer Pai
V$GREF/ARE.02	Androgene receptor binding site, IR3 sites	BreastEndocrine SystemLeydig CellsOvaryProstateTestisUrogenital System	0.89	-4671	-4653	-4662	+	-1	0.934	0.044	0		gcagggccttctGTCCtca	pair1
V$GREF/ARE.03	Androgene receptor binding site, IR3 sites	BreastEndocrine SystemLeydig CellsOvaryProstateTestisUrogenital System	0.87	-4671	-4653	-4662	+	-1	0.896	0.026	0		gcagggccttctGTCCtca	pair1
V$GREF/GRE.01	Glucocorticoid receptor, C2C2 zinc finger protein binds glucocorticoid dependent to GREs, IR3 sites	BreastEndocrine SystemLeydig CellsOvaryProstateTestisUrogenital System	0.85	-4671	-4653	-4662	+	-1	0.868	0.018	0		gcagggccttctGTCCtca	pair1
V$GREF/GRE.03	Glucocorticoid receptor, IR3 sites	BreastEndocrine SystemLeydig CellsOvaryProstateTestisUrogenital System	0.88	-4671	-4653	-4662	+	-1	0.866	-0.014	0		gcagggccttcTGTCctca	pair1
V$GREF/GRE.02	Glucocorticoid receptor, IR3 sites	BreastEndocrine SystemLeydig CellsOvaryProstateTestisUrogenital System	0.82	-4671	-4653	-4662	-	-1	0.836	0.016	0		tgaGGACagaaggccctgc	pair1
V$GREF/PRE.01	Progesterone receptor binding site, IR3 sites	BreastEndocrine SystemLeydig CellsOvaryProstateTestisUrogenital System	0.84	-4671	-4653	-4662	+	-1	0.788	-0.052	0		gcagggccttcTGTCctca	pair1
V$GREF/ARE.01	Androgene receptor binding site, IR3 sites	BreastEndocrine SystemLeydig CellsOvaryProstateTestisUrogenital System	0.8	-4671	-4653	-4662	+	-1	0.782	-0.018	0		gcagggccttcTGTCctca	pair1
V$GREF/ARE.02	Androgene receptor binding site, IR3 sites	BreastEndocrine SystemLeydig CellsOvaryProstateTestisUrogenital System	0.89	-4671	-4653	-4662	-	-1	0.73	-0.16	0		tgaggacagaagGCCCtgc	pair1
V$GREF/ARE.03	Androgene receptor binding site, IR3 sites	BreastEndocrine SystemLeydig CellsOvaryProstateTestisUrogenital System	0.87	-4671	-4653	-4662	-	-1	0.717	-0.153	0		tgaggacagaagGCCCtgc	pair1
V$GREF/GRE.02	Glucocorticoid receptor, IR3 sites	BreastEndocrine SystemLeydig CellsOvaryProstateTestisUrogenital System	0.82	-4671	-4653	-4662	+	-1	0.697	-0.123	0		gcaGGGCcttctgtcctca	pair1
V$GREF/GRE.01	Glucocorticoid receptor, C2C2 zinc finger protein binds glucocorticoid dependent to GREs, IR3 sites	BreastEndocrine SystemLeydig CellsOvaryProstateTestisUrogenital System	0.85	-4671	-4653	-4662	-	-1	0.667	-0.183	0		tgaggacagaagGCCCtgc	pair1
V$GREF/ARE.01	Androgene receptor binding site, IR3 sites	BreastEndocrine SystemLeydig CellsOvaryProstateTestisUrogenital System	0.8	-4671	-4653	-4662	-	-1	0.609	-0.191	0		tgaggacagaaGGCCctgc	pair1
V$NGRE/IR1_NGRE.01	Repressive binding sites for glucocorticoid receptor (IR1)	Adrenal GlandsBrainCentral Nervous SystemEndocrine SystemNervous System	0.81	-4328	-4314	-4321	-	-1	0.91	0.1	0		ggcttccaGGAGaac	pair2
V$NGRE/IR2_NGRE.01	Repressive binding sites for glucocorticoid receptor (IR2)	Adrenal GlandsBrainCentral Nervous SystemEndocrine SystemNervous System	0.8	-4328	-4314	-4321	-	-1	0.877	0.077	0		ggCTTCcaggagaac	pair2
V$NGRE/IR1_NGRE.01	Repressive binding sites for glucocorticoid receptor (IR1)	Adrenal GlandsBrainCentral Nervous SystemEndocrine SystemNervous System	0.81	-4328	-4314	-4321	+	-1	0.78	-0.03	0		gttctcctGGAAgcc	pair2
V$GREF/GRE.03	Glucocorticoid receptor, IR3 sites	BreastEndocrine SystemLeydig CellsOvaryProstateTestisUrogenital System	0.88	-4174	-4156	-4165	-	-1	0.876	-0.004	0		tgcagacacctTGTCccaa	pair3
V$GREF/ARE.02	Androgene receptor binding site, IR3 sites	BreastEndocrine SystemLeydig CellsOvaryProstateTestisUrogenital System	0.89	-4174	-4156	-4165	-	-1	0.87	-0.02	0		tgcagacaccttGTCCcaa	pair3
V$GREF/ARE.03	Androgene receptor binding site, IR3 sites	BreastEndocrine SystemLeydig CellsOvaryProstateTestisUrogenital System	0.87	-4174	-4156	-4165	-	-1	0.853	-0.017	0		tgcagacaccttGTCCcaa	pair3
V$GREF/GRE.02	Glucocorticoid receptor, IR3 sites	BreastEndocrine SystemLeydig CellsOvaryProstateTestisUrogenital System	0.82	-4174	-4156	-4165	+	-1	0.852	0.032	0		ttgGGACaaggtgtctgca	pair3
V$GREF/GRE.02	Glucocorticoid receptor, IR3 sites	BreastEndocrine SystemLeydig CellsOvaryProstateTestisUrogenital System	0.82	-4174	-4156	-4165	-	-1	0.752	-0.068	0		tgcAGACaccttgtcccaa	pair3
V$GREF/ARE.03	Androgene receptor binding site, IR3 sites	BreastEndocrine SystemLeydig CellsOvaryProstateTestisUrogenital System	0.87	-4174	-4156	-4165	+	-1	0.728	-0.142	0		ttgggacaaggtGTCTgca	pair3
V$GREF/GRE.03	Glucocorticoid receptor, IR3 sites	BreastEndocrine SystemLeydig CellsOvaryProstateTestisUrogenital System	0.88	-4174	-4156	-4165	+	-1	0.719	-0.161	0		ttgggacaaggTGTCtgca	pair3
V$GREF/ARE.02	Androgene receptor binding site, IR3 sites	BreastEndocrine SystemLeydig CellsOvaryProstateTestisUrogenital System	0.89	-4174	-4156	-4165	+	-1	0.712	-0.178	0		ttgggacaaggtGTCTgca	pair3
V$GREF/GRE.01	Glucocorticoid receptor, C2C2 zinc finger protein binds glucocorticoid dependent to GREs, IR3 sites	BreastEndocrine SystemLeydig CellsOvaryProstateTestisUrogenital System	0.85	-4174	-4156	-4165	-	-1	0.688	-0.162	0		tgcagacaccttGTCCcaa	pair3
V$GREF/PRE.01	Progesterone receptor binding site, IR3 sites	BreastEndocrine SystemLeydig CellsOvaryProstateTestisUrogenital System	0.84	-4174	-4156	-4165	-	-1	0.667	-0.173	0		tgcagacacctTGTCccaa	pair3
V$GREF/ARE.01	Androgene receptor binding site, IR3 sites	BreastEndocrine SystemLeydig CellsOvaryProstateTestisUrogenital System	0.8	-4174	-4156	-4165	-	-1	0.638	-0.162	0		tgcagacacctTGTCccaa	pair3
V$GREF/ARE.01	Androgene receptor binding site, IR3 sites	BreastEndocrine SystemLeydig CellsOvaryProstateTestisUrogenital System	0.8	-4174	-4156	-4165	+	-1	0.636	-0.164	0		ttgggacaaggTGTCtgca	pair3
V$GREF/ARE.03	Androgene receptor binding site, IR3 sites	BreastEndocrine SystemLeydig CellsOvaryProstateTestisUrogenital System	0.87	-3957	-3939	-3948	-	-1	0.901	0.031	0		ggggccctgactGTGCttg	pair4
V$GREF/GRE.01	Glucocorticoid receptor, C2C2 zinc finger protein binds glucocorticoid dependent to GREs, IR3 sites	BreastEndocrine SystemLeydig CellsOvaryProstateTestisUrogenital System	0.85	-3957	-3939	-3948	-	-1	0.889	0.039	0		ggggccctgactGTGCttg	pair4
V$GREF/ARE.02	Androgene receptor binding site, IR3 sites	BreastEndocrine SystemLeydig CellsOvaryProstateTestisUrogenital System	0.89	-3957	-3939	-3948	-	-1	0.885	-0.005	0		ggggccctgactGTGCttg	pair4
V$GREF/GRE.03	Glucocorticoid receptor, IR3 sites	BreastEndocrine SystemLeydig CellsOvaryProstateTestisUrogenital System	0.88	-3957	-3939	-3948	-	-1	0.874	-0.006	0		ggggccctgacTGTGcttg	pair4
V$GREF/GRE.02	Glucocorticoid receptor, IR3 sites	BreastEndocrine SystemLeydig CellsOvaryProstateTestisUrogenital System	0.82	-3957	-3939	-3948	+	-1	0.819	-0.001	0		caaGCACagtcagggcccc	pair4
V$GREF/PRE.01	Progesterone receptor binding site, IR3 sites	BreastEndocrine SystemLeydig CellsOvaryProstateTestisUrogenital System	0.84	-3957	-3939	-3948	-	-1	0.804	-0.036	0		ggggccctgacTGTGcttg	pair4
V$GREF/ARE.01	Androgene receptor binding site, IR3 sites	BreastEndocrine SystemLeydig CellsOvaryProstateTestisUrogenital System	0.8	-3957	-3939	-3948	-	-1	0.787	-0.013	0		ggggccctgacTGTGcttg	pair4
V$GREF/ARE.02	Androgene receptor binding site, IR3 sites	BreastEndocrine SystemLeydig CellsOvaryProstateTestisUrogenital System	0.89	-3957	-3939	-3948	+	-1	0.718	-0.172	0		caagcacagtcaGGGCccc	pair4
V$GREF/GRE.02	Glucocorticoid receptor, IR3 sites	BreastEndocrine SystemLeydig CellsOvaryProstateTestisUrogenital System	0.82	-3957	-3939	-3948	-	-1	0.683	-0.137	0		gggGCCCtgactgtgcttg	pair4
V$GREF/GRE.03	Glucocorticoid receptor, IR3 sites	BreastEndocrine SystemLeydig CellsOvaryProstateTestisUrogenital System	0.88	-3840	-3822	-3831	-	-1	0.887	0.007	0		cctacacacccTGTCcctg	pair5
V$GREF/ARE.02	Androgene receptor binding site, IR3 sites	BreastEndocrine SystemLeydig CellsOvaryProstateTestisUrogenital System	0.89	-3840	-3822	-3831	-	-1	0.879	-0.011	0		cctacacaccctGTCCctg	pair5
V$GREF/GRE.02	Glucocorticoid receptor, IR3 sites	BreastEndocrine SystemLeydig CellsOvaryProstateTestisUrogenital System	0.82	-3840	-3822	-3831	+	-1	0.868	0.048	0		cagGGACagggtgtgtagg	pair5
V$GREF/ARE.03	Androgene receptor binding site, IR3 sites	BreastEndocrine SystemLeydig CellsOvaryProstateTestisUrogenital System	0.87	-3840	-3822	-3831	-	-1	0.86	-0.01	0		cctacacaccctGTCCctg	pair5
V$GREF/GRE.02	Glucocorticoid receptor, IR3 sites	BreastEndocrine SystemLeydig CellsOvaryProstateTestisUrogenital System	0.82	-3840	-3822	-3831	-	-1	0.761	-0.059	0		cctACACaccctgtccctg	pair5
V$GREF/ARE.03	Androgene receptor binding site, IR3 sites	BreastEndocrine SystemLeydig CellsOvaryProstateTestisUrogenital System	0.87	-3840	-3822	-3831	+	-1	0.736	-0.134	0		cagggacagggtGTGTagg	pair5
V$GREF/GRE.03	Glucocorticoid receptor, IR3 sites	BreastEndocrine SystemLeydig CellsOvaryProstateTestisUrogenital System	0.88	-3840	-3822	-3831	+	-1	0.723	-0.157	0		cagggacagggTGTGtagg	pair5
V$GREF/GRE.01	Glucocorticoid receptor, C2C2 zinc finger protein binds glucocorticoid dependent to GREs, IR3 sites	BreastEndocrine SystemLeydig CellsOvaryProstateTestisUrogenital System	0.85	-3840	-3822	-3831	-	-1	0.705	-0.145	0		cctacacaccctGTCCctg	pair5
V$GREF/ARE.02	Androgene receptor binding site, IR3 sites	BreastEndocrine SystemLeydig CellsOvaryProstateTestisUrogenital System	0.89	-3840	-3822	-3831	+	-1	0.695	-0.195	0		cagggacagggtGTGTagg	pair5
V$GREF/PRE.01	Progesterone receptor binding site, IR3 sites	BreastEndocrine SystemLeydig CellsOvaryProstateTestisUrogenital System	0.84	-3840	-3822	-3831	-	-1	0.669	-0.171	0		cctacacacccTGTCcctg	pair5
V$GREF/ARE.01	Androgene receptor binding site, IR3 sites	BreastEndocrine SystemLeydig CellsOvaryProstateTestisUrogenital System	0.8	-3840	-3822	-3831	-	-1	0.641	-0.159	0		cctacacacccTGTCcctg	pair5
V$GREF/ARE.01	Androgene receptor binding site, IR3 sites	BreastEndocrine SystemLeydig CellsOvaryProstateTestisUrogenital System	0.8	-3840	-3822	-3831	+	-1	0.638	-0.162	0		cagggacagggTGTGtagg	pair5
V$GREF/ARE.02	Androgene receptor binding site, IR3 sites	BreastEndocrine SystemLeydig CellsOvaryProstateTestisUrogenital System	0.89	-2972	-2954	-2963	-	-1	0.87	-0.02	0		ctggctcactctGTCCctc	pair6a
V$GREF/GRE.03	Glucocorticoid receptor, IR3 sites	BreastEndocrine SystemLeydig CellsOvaryProstateTestisUrogenital System	0.88	-2972	-2954	-2963	-	-1	0.869	-0.011	0		ctggctcactcTGTCcctc	pair6a
V$GREF/ARE.03	Androgene receptor binding site, IR3 sites	BreastEndocrine SystemLeydig CellsOvaryProstateTestisUrogenital System	0.87	-2972	-2954	-2963	-	-1	0.86	-0.01	0		ctggctcactctGTCCctc	pair6a
V$GREF/GRE.02	Glucocorticoid receptor, IR3 sites	BreastEndocrine SystemLeydig CellsOvaryProstateTestisUrogenital System	0.82	-2972	-2954	-2963	+	-1	0.828	0.008	0		gagGGACagagtgagccag	pair6a
V$GREF/GRE.02	Glucocorticoid receptor, IR3 sites	BreastEndocrine SystemLeydig CellsOvaryProstateTestisUrogenital System	0.82	-2972	-2954	-2963	-	-1	0.795	-0.025	0		ctgGCTCactctgtccctc	pair6a
V$GREF/ARE.02	Androgene receptor binding site, IR3 sites	BreastEndocrine SystemLeydig CellsOvaryProstateTestisUrogenital System	0.89	-2972	-2954	-2963	+	-1	0.76	-0.13	0		gagggacagagtGAGCcag	pair6a
V$GREF/GRE.01	Glucocorticoid receptor, C2C2 zinc finger protein binds glucocorticoid dependent to GREs, IR3 sites	BreastEndocrine SystemLeydig CellsOvaryProstateTestisUrogenital System	0.85	-2972	-2954	-2963	-	-1	0.754	-0.096	0		ctggctcactctGTCCctc	pair6a
V$GREF/GRE.03	Glucocorticoid receptor, IR3 sites	BreastEndocrine SystemLeydig CellsOvaryProstateTestisUrogenital System	0.88	-2972	-2954	-2963	+	-1	0.744	-0.136	0		gagggacagagTGAGccag	pair6a
V$GREF/ARE.03	Androgene receptor binding site, IR3 sites	BreastEndocrine SystemLeydig CellsOvaryProstateTestisUrogenital System	0.87	-2972	-2954	-2963	+	-1	0.719	-0.151	0		gagggacagagtGAGCcag	pair6a
V$GREF/ARE.01	Androgene receptor binding site, IR3 sites	BreastEndocrine SystemLeydig CellsOvaryProstateTestisUrogenital System	0.8	-2972	-2954	-2963	-	-1	0.683	-0.117	0		ctggctcactcTGTCcctc	pair6a
V$GREF/PRE.01	Progesterone receptor binding site, IR3 sites	BreastEndocrine SystemLeydig CellsOvaryProstateTestisUrogenital System	0.84	-2972	-2954	-2963	-	-1	0.67	-0.17	0		ctggctcactcTGTCcctc	pair6a
V$GREF/ARE.01	Androgene receptor binding site, IR3 sites	BreastEndocrine SystemLeydig CellsOvaryProstateTestisUrogenital System	0.8	-2972	-2954	-2963	+	-1	0.643	-0.157	0		gagggacagagTGAGccag	pair6a
V$GREF/ARE.02	Androgene receptor binding site, IR3 sites	BreastEndocrine SystemLeydig CellsOvaryProstateTestisUrogenital System	0.89	-2934	-2916	-2925	+	-1	0.897	0.007	0		ctcaatcatccaGTGCtga	pair6b
V$GREF/ARE.03	Androgene receptor binding site, IR3 sites	BreastEndocrine SystemLeydig CellsOvaryProstateTestisUrogenital System	0.87	-2934	-2916	-2925	+	-1	0.811	-0.059	0		ctcaatcatccaGTGCtga	pair6b
V$GREF/GRE.01	Glucocorticoid receptor, C2C2 zinc finger protein binds glucocorticoid dependent to GREs, IR3 sites	BreastEndocrine SystemLeydig CellsOvaryProstateTestisUrogenital System	0.85	-2934	-2916	-2925	+	-1	0.736	-0.114	0		ctcaatcatccaGTGCtga	pair6b
V$GREF/GRE.03	Glucocorticoid receptor, IR3 sites	BreastEndocrine SystemLeydig CellsOvaryProstateTestisUrogenital System	0.88	-2934	-2916	-2925	+	-1	0.693	-0.187	0		ctcaatcatccAGTGctga	pair6b
V$GREF/GRE.02	Glucocorticoid receptor, IR3 sites	BreastEndocrine SystemLeydig CellsOvaryProstateTestisUrogenital System	0.82	-2934	-2916	-2925	-	-1	0.691	-0.129	0		tcaGCACtggatgattgag	pair6b
V$GREF/GRE.02	Glucocorticoid receptor, IR3 sites	BreastEndocrine SystemLeydig CellsOvaryProstateTestisUrogenital System	0.82	-2934	-2916	-2925	+	-1	0.628	-0.192	0		ctcAATCatccagtgctga	pair6b
V$NGRE/IR2_NGRE.01	Repressive binding sites for glucocorticoid receptor (IR2)	Adrenal GlandsBrainCentral Nervous SystemEndocrine SystemNervous System	0.8	-2702	-2688	-2695	+	-1	0.873	0.073	0		gcCTCCaggaagagc	pair7a
V$NGRE/IR2_NGRE.01	Repressive binding sites for glucocorticoid receptor (IR2)	Adrenal GlandsBrainCentral Nervous SystemEndocrine SystemNervous System	0.8	-2614	-2600	-2607	+	-1	0.883	0.083	0		tcCTCCagggacacc	pair7b
V$NGRE/IR1_NGRE.01	Repressive binding sites for glucocorticoid receptor (IR1)	Adrenal GlandsBrainCentral Nervous SystemEndocrine SystemNervous System	0.81	-2614	-2600	-2607	+	-1	0.616	-0.194	0		tcctccagGGACacc	pair7b
V$NGRE/IR1_NGRE.01	Repressive binding sites for glucocorticoid receptor (IR1)	Adrenal GlandsBrainCentral Nervous SystemEndocrine SystemNervous System	0.81	-2154	-2140	-2147	+	-1	0.814	0.004	0		ggcctcttGGAGgaa	pair8
V$NGRE/IR1_NGRE.01	Repressive binding sites for glucocorticoid receptor (IR1)	Adrenal GlandsBrainCentral Nervous SystemEndocrine SystemNervous System	0.81	-2154	-2140	-2147	-	-1	0.757	-0.053	0		ttcctccaAGAGgcc	pair8
V$NGRE/IR2_NGRE.01	Repressive binding sites for glucocorticoid receptor (IR2)	Adrenal GlandsBrainCentral Nervous SystemEndocrine SystemNervous System	0.8	-2154	-2140	-2147	+	-1	0.69	-0.11	0		ggCCTCttggaggaa	pair8
V$GREF/PRE.01	Progesterone receptor binding site, IR3 sites	BreastEndocrine SystemLeydig CellsOvaryProstateTestisUrogenital System	0.84	-135	-117	-126	+	-1	0.901	0.061	0		caatcaaatggTGTTctgc	pair9
V$GREF/GRE.03	Glucocorticoid receptor, IR3 sites	BreastEndocrine SystemLeydig CellsOvaryProstateTestisUrogenital System	0.88	-135	-117	-126	+	-1	0.892	0.012	0		caatcaaatggTGTTctgc	pair9
V$GREF/ARE.03	Androgene receptor binding site, IR3 sites	BreastEndocrine SystemLeydig CellsOvaryProstateTestisUrogenital System	0.87	-135	-117	-126	+	-1	0.858	-0.012	0		caatcaaatggtGTTCtgc	pair9
V$GREF/GRE.01	Glucocorticoid receptor, C2C2 zinc finger protein binds glucocorticoid dependent to GREs, IR3 sites	BreastEndocrine SystemLeydig CellsOvaryProstateTestisUrogenital System	0.85	-135	-117	-126	+	-1	0.839	-0.011	0		caatcaaatggtGTTCtgc	pair9
V$GREF/ARE.01	Androgene receptor binding site, IR3 sites	BreastEndocrine SystemLeydig CellsOvaryProstateTestisUrogenital System	0.8	-135	-117	-126	+	-1	0.808	0.008	0		caatcaaatggTGTTctgc	pair9
V$GREF/GRE.02	Glucocorticoid receptor, IR3 sites	BreastEndocrine SystemLeydig CellsOvaryProstateTestisUrogenital System	0.82	-135	-117	-126	-	-1	0.767	-0.053	0		gcaGAACaccatttgattg	pair9
V$GREF/ARE.02	Androgene receptor binding site, IR3 sites	BreastEndocrine SystemLeydig CellsOvaryProstateTestisUrogenital System	0.89	-135	-117	-126	+	-1	0.758	-0.132	0		caatcaaatggtGTTCtgc	pair9
V$GREF/GRE.02	Glucocorticoid receptor, IR3 sites	BreastEndocrine SystemLeydig CellsOvaryProstateTestisUrogenital System	0.82	-135	-117	-126	+	-1	0.711	-0.109	0		caaTCAAatggtgttctgc	pair9
V$GREF/ARE.01	Androgene receptor binding site, IR3 sites	BreastEndocrine SystemLeydig CellsOvaryProstateTestisUrogenital System	0.8	-135	-117	-126	-	-1	0.627	-0.173	0		gcagaacaccaTTTGattg	pair9
V$NGRE/IR2_NGRE.01	Repressive binding sites for glucocorticoid receptor (IR2)	Adrenal GlandsBrainCentral Nervous SystemEndocrine SystemNervous System	0.8	5	19	12	+	-1	0.889	0.089	0		ccCTCCctggacacc	pair10
V$NGRE/IR1_NGRE.01	Repressive binding sites for glucocorticoid receptor (IR1)	Adrenal GlandsBrainCentral Nervous SystemEndocrine SystemNervous System	0.81	5	19	12	+	-1	0.709	-0.101	0		ccctccctGGACacc	pair10
V$GREF/PRE.01	Progesterone receptor binding site, IR3 sites	BreastEndocrine SystemLeydig CellsOvaryProstateTestisUrogenital System	0.84	742	760	751	+	-1	0.884	0.044	0		ttgggctgaatTGTTcttt	pair11
V$GREF/GRE.01	Glucocorticoid receptor, C2C2 zinc finger protein binds glucocorticoid dependent to GREs, IR3 sites	BreastEndocrine SystemLeydig CellsOvaryProstateTestisUrogenital System	0.85	742	760	751	+	-1	0.861	0.011	0		ttgggctgaattGTTCttt	pair11
V$GREF/GRE.03	Glucocorticoid receptor, IR3 sites	BreastEndocrine SystemLeydig CellsOvaryProstateTestisUrogenital System	0.88	742	760	751	+	-1	0.86	-0.02	0		ttgggctgaatTGTTcttt	pair11
V$GREF/ARE.01	Androgene receptor binding site, IR3 sites	BreastEndocrine SystemLeydig CellsOvaryProstateTestisUrogenital System	0.8	742	760	751	+	-1	0.838	0.038	0		ttgggctgaatTGTTcttt	pair11
V$GREF/ARE.03	Androgene receptor binding site, IR3 sites	BreastEndocrine SystemLeydig CellsOvaryProstateTestisUrogenital System	0.87	742	760	751	+	-1	0.825	-0.045	0		ttgggctgaattGTTCttt	pair11
V$GREF/GRE.02	Glucocorticoid receptor, IR3 sites	BreastEndocrine SystemLeydig CellsOvaryProstateTestisUrogenital System	0.82	742	760	751	-	-1	0.681	-0.139	0		aaaGAACaattcagcccaa	pair11
V$GREF/PRE.01	Progesterone receptor binding site, IR3 sites	BreastEndocrine SystemLeydig CellsOvaryProstateTestisUrogenital System	0.84	1074	1092	1083	+	-1	0.849	0.009	0		cagcccagaatTGTTcttg	pair12
V$GREF/GRE.01	Glucocorticoid receptor, C2C2 zinc finger protein binds glucocorticoid dependent to GREs, IR3 sites	BreastEndocrine SystemLeydig CellsOvaryProstateTestisUrogenital System	0.85	1074	1092	1083	+	-1	0.822	-0.028	0		cagcccagaattGTTCttg	pair12
V$GREF/GRE.03	Glucocorticoid receptor, IR3 sites	BreastEndocrine SystemLeydig CellsOvaryProstateTestisUrogenital System	0.88	1074	1092	1083	+	-1	0.82	-0.06	0		cagcccagaatTGTTcttg	pair12
V$GREF/ARE.03	Androgene receptor binding site, IR3 sites	BreastEndocrine SystemLeydig CellsOvaryProstateTestisUrogenital System	0.87	1074	1092	1083	+	-1	0.769	-0.101	0		cagcccagaattGTTCttg	pair12
V$GREF/ARE.01	Androgene receptor binding site, IR3 sites	BreastEndocrine SystemLeydig CellsOvaryProstateTestisUrogenital System	0.8	1074	1092	1083	+	-1	0.745	-0.055	0		cagcccagaatTGTTcttg	pair12
V$NGRE/IR2_NGRE.01	Repressive binding sites for glucocorticoid receptor (IR2)	Adrenal GlandsBrainCentral Nervous SystemEndocrine SystemNervous System	0.8	1325	1339	1332	+	-1	0.875	0.075	0		ctCTCCagggtgata	pair13a
V$NGRE/IR2_NGRE.01	Repressive binding sites for glucocorticoid receptor (IR2)	Adrenal GlandsBrainCentral Nervous SystemEndocrine SystemNervous System	0.8	1398	1412	1405	+	-1	0.803	0.003	0		ctCTCCtagggggag	pair13b
V$GREF/ARE.02	Androgene receptor binding site, IR3 sites	BreastEndocrine SystemLeydig CellsOvaryProstateTestisUrogenital System	0.89	2445	2463	2454	+	-1	0.866	-0.024	0		tgccaactggctGTGCctc	pair14
V$GREF/GRE.02	Glucocorticoid receptor, IR3 sites	BreastEndocrine SystemLeydig CellsOvaryProstateTestisUrogenital System	0.82	2445	2463	2454	-	-1	0.866	0.046	0		gagGCACagccagttggca	pair14
V$GREF/GRE.03	Glucocorticoid receptor, IR3 sites	BreastEndocrine SystemLeydig CellsOvaryProstateTestisUrogenital System	0.88	2445	2463	2454	+	-1	0.862	-0.018	0		tgccaactggcTGTGcctc	pair14
V$GREF/ARE.03	Androgene receptor binding site, IR3 sites	BreastEndocrine SystemLeydig CellsOvaryProstateTestisUrogenital System	0.87	2445	2463	2454	+	-1	0.837	-0.033	0		tgccaactggctGTGCctc	pair14
V$GREF/ARE.02	Androgene receptor binding site, IR3 sites	BreastEndocrine SystemLeydig CellsOvaryProstateTestisUrogenital System	0.89	2445	2463	2454	-	-1	0.693	-0.197	0		gaggcacagccaGTTGgca	pair14
V$GREF/ARE.03	Androgene receptor binding site, IR3 sites	BreastEndocrine SystemLeydig CellsOvaryProstateTestisUrogenital System	0.87	2445	2463	2454	-	-1	0.689	-0.181	0		gaggcacagccaGTTGgca	pair14
V$GREF/GRE.01	Glucocorticoid receptor, C2C2 zinc finger protein binds glucocorticoid dependent to GREs, IR3 sites	BreastEndocrine SystemLeydig CellsOvaryProstateTestisUrogenital System	0.85	2445	2463	2454	+	-1	0.672	-0.178	0		tgccaactggctGTGCctc	pair14
V$GREF/PRE.01	Progesterone receptor binding site, IR3 sites	BreastEndocrine SystemLeydig CellsOvaryProstateTestisUrogenital System	0.84	2445	2463	2454	+	-1	0.671	-0.169	0		tgccaactggcTGTGcctc	pair14
V$GREF/GRE.02	Glucocorticoid receptor, IR3 sites	BreastEndocrine SystemLeydig CellsOvaryProstateTestisUrogenital System	0.82	2445	2463	2454	+	-1	0.649	-0.171	0		tgcCAACtggctgtgcctc	pair14
V$GREF/ARE.01	Androgene receptor binding site, IR3 sites	BreastEndocrine SystemLeydig CellsOvaryProstateTestisUrogenital System	0.8	2445	2463	2454	+	-1	0.628	-0.172	0		tgccaactggcTGTGcctc	pair14
V$GREF/ARE.01	Androgene receptor binding site, IR3 sites	BreastEndocrine SystemLeydig CellsOvaryProstateTestisUrogenital System	0.8	2445	2463	2454	-	-1	0.624	-0.176	0		gaggcacagccAGTTggca	pair14
V$GREF/ARE.03	Androgene receptor binding site, IR3 sites	BreastEndocrine SystemLeydig CellsOvaryProstateTestisUrogenital System	0.87	3068	3086	3077	+	-1	0.892	0.022	0		tctgacccccctGTCCtcc	pair15
V$GREF/ARE.02	Androgene receptor binding site, IR3 sites	BreastEndocrine SystemLeydig CellsOvaryProstateTestisUrogenital System	0.89	3068	3086	3077	+	-1	0.881	-0.009	0		tctgacccccctGTCCtcc	pair15
V$GREF/GRE.03	Glucocorticoid receptor, IR3 sites	BreastEndocrine SystemLeydig CellsOvaryProstateTestisUrogenital System	0.88	3068	3086	3077	+	-1	0.863	-0.017	0		tctgaccccccTGTCctcc	pair15
V$GREF/GRE.01	Glucocorticoid receptor, C2C2 zinc finger protein binds glucocorticoid dependent to GREs, IR3 sites	BreastEndocrine SystemLeydig CellsOvaryProstateTestisUrogenital System	0.85	3068	3086	3077	+	-1	0.847	-0.003	0		tctgacccccctGTCCtcc	pair15
V$GREF/GRE.02	Glucocorticoid receptor, IR3 sites	BreastEndocrine SystemLeydig CellsOvaryProstateTestisUrogenital System	0.82	3068	3086	3077	-	-1	0.845	0.025	0		ggaGGACaggggggtcaga	pair15
V$GREF/ARE.01	Androgene receptor binding site, IR3 sites	BreastEndocrine SystemLeydig CellsOvaryProstateTestisUrogenital System	0.8	3068	3086	3077	+	-1	0.765	-0.035	0		tctgaccccccTGTCctcc	pair15
V$GREF/PRE.01	Progesterone receptor binding site, IR3 sites	BreastEndocrine SystemLeydig CellsOvaryProstateTestisUrogenital System	0.84	3068	3086	3077	+	-1	0.755	-0.085	0		tctgaccccccTGTCctcc	pair15
V$GREF/ARE.03	Androgene receptor binding site, IR3 sites	BreastEndocrine SystemLeydig CellsOvaryProstateTestisUrogenital System	0.87	3068	3086	3077	-	-1	0.671	-0.199	0		ggaggacaggggGGTCaga	pair15
V$GREF/GRE.02	Glucocorticoid receptor, IR3 sites	BreastEndocrine SystemLeydig CellsOvaryProstateTestisUrogenital System	0.82	3068	3086	3077	+	-1	0.668	-0.152	0		tctGACCcccctgtcctcc	pair15
V$GREF/ARE.01	Androgene receptor binding site, IR3 sites	BreastEndocrine SystemLeydig CellsOvaryProstateTestisUrogenital System	0.8	3068	3086	3077	-	-1	0.632	-0.168	0		ggaggacagggGGGTcaga	pair15
