# Supplementary material for: HIV-1 Vpr suppresses expression of the thiazide-sensitive sodium chloride co-transporter in the distal convoluted tubule
Source: PLoS One. 2022 Sep 21;17(9):e0273313. doi: 10.1371/journal.pone.0273313 (PMC9491550; doi:10.1371/journal.pone.0273313)
Supplement: Supplementary file 5 [file pone.0273313.s007.docx]

			Sequence		Distance from 1st exon		
**Pair 1**	**Forward**	5'-	GCTGAGTTGCCTCTGTAGCC	-3'			
	**Reverse**	5'-	AGATGCAGGTCCTGAAATGG	-3'			
	**Target motif**	5'-	GCAGGGCCTTCTGTCCTCA	-3'	-4671	~	-4653
**Pair 2**	**Forward**	5'-	TTGCTCCAGGTACACAGAACC	-3'			
	**Reverse**	5'-	CACAGTCCCTACCTTTCAGGA	-3'			
	**Target motif**	5'-	GTTCTCCTGGAAGCC	-3'	-4328		-4314
**Pair 3**	**Forward**	5'-	AATGCAGGCTTCCCCTTC	-3'			
	**Reverse**	5'-	GTCCCCTCGAAGGACTCATC	-3'			
	**Target motif**	5'-	TTGGGACAAGGTGTCTGCA	-3'	-4174		-4156
**Pair 4**	**Forward**	5'-	AGCTGTCTAGGCCAGGGTTT	-3'			
	**Reverse**	5'-	GTGGTCCTGGGCAGAGTG	-3'			
	**Target motif**	5'-	CAAGCACAGTCAGGGCCCC	-3'	-3957		-3939
**Pair 5**	**Forward**	5'-	CTCAGGCTCCTTCCTCTGTG	-3'			
	**Reverse**	5'-	GCTGCCCTTTATCTGCTCAC	-3'			
	**Target motif**	5'-	CAGGGACAGGGTGTGTAGG	-3'	-3840		-3822
**Pair 6**	**Forward**	5'-	ACAGCTGGGTGGGTACACCT	-3'			
	**Reverse**	5'-	ATCCTTAGGGCTCCATGACC	-3'			
	**Target**	5'-	GAGGGACAGAGTGAGCCAG	-3'	-2972		-2954
	**motif**	5'-	CTCAATCATCCAGTGCTGA	-3'	-2934		-2916
**Pair 7**	**Forward**	5'-	CCCAAGGATGACCAGAGCTA	-3'			
	**Reverse**	5'-	AGGGTAGCAATGGGAGAGGT	-3'			
	**Target**	5'-	GCCTCCAGGAAGAG	-3'	-2702		-2689
	**motif**	5'-	TCCTCCAGGGACACC	-3'	-2614		-2600
**Pair 8**	**Forward**	5'-	GGCAGGGTGTCAGCTCTTTA	-3'			
	**Reverse**	5'-	TGTTGTGAGGGCTCTCAGTG	-3'			
	**Target motif**	5'-	GGCCTCTTGGAGGAA	-3'	-2154		-2140
**Pair 9**	**Forward**	5'-	AGCCCTCCAGATCCAAGG	-3'			
	**Reverse**	5'-	CAGGGAGGTTGGCCTAAA	-3'			
	**Target motif**	5'-	CAATCAAATGGTGTTCTGC	-3'	-135		-117
	**Mutant sequence ***	5'-	ATATGATATGAAATTAGGG	-3'	-135		-117
**Pair 10**	**Forward**	5'-	TGCAGACGCAGCCTATAAAA	-3'			
	**Reverse**	5'-	CGTCTCTGTTGTGGGCAGT	-3'			
	**Target motif**	5'-	CCCTCCCTGGACACC	-3'	+5		+19
	**Mutant sequence ***	5'-	GCGCTCTATGTGACG	-3'	+5		+19
**Pair 11**	**Forward**	5'-	CTGGCCTCACTTTTCCCATA	-3'			
	**Reverse**	5'-	GCGACAGAGCCAGACTCC	-3'			
	**Target motif**	5'-	TTGGGCTGAATTGTTCTTT	-3'	+742		+760
**Pair 12**	**Forward**	5'-	TGCTGGAATTACAGGCATGA	-3'			
	**Reverse**	5'-	GAGAAAAGGCCTGCGAATTT	-3'			
	**Target motif**	5'-	CAGCCCAGAATTGTTCTTG	-3'	+1074		+1092
**Pair 13**	**Forward**	5'-	ACCAACTATGTGGCCTTGGA	-3'			
	**Reverse**	5'-	GAGGTGGAGGTTGCAGTGAT	-3'			
	**Target**	5'-	CTCTCCAGGGTGATA	-3'	+1325		+1339
	**motif**	5'-	CTCTCCTAGGGGGAG	-3'	+1398		+1412
**Pair 14**	**Forward**	5'-	AGAAGGCTTTAGCCCTGCTC	-3'			
	**Reverse**	5'-	TCGTGGTTCAGATGGAGAAA	-3'			
	**Target**	5'-	CTGCTCCGGGAGGCA	-3'	2405		2419
	**motif**	5'-	TGCCAACTGGCTGTGCCTC	-3'	+2445		+2463
**Pair 15**	**Forward**	5'-	TAGCAAGGGACAGGGACTTG	-3'			
	**Reverse**	5'-	AATGTTGAGCATGCAACGAA	-3'			
	**Target motif**	5'-	TCTGACCCCCCTGTCCTCC	-3'	+3068		+3086
Note *			Mutants				
