# Supplementary material for: HIV-1 Vpr suppresses expression of the thiazide-sensitive sodium chloride co-transporter in the distal convoluted tubule
Source: PLoS One. 2022 Sep 21;17(9):e0273313. doi: 10.1371/journal.pone.0273313 (PMC9491550; doi:10.1371/journal.pone.0273313)
Supplement: Attachment — Submitted filename: 04_Response to reviewers_07132022.docx [file pone.0273313.s010.docx]

PONE-D-22-10698

HIV-1 Vpr suppresses expression of the thiazide-sensitive sodium chloride co-transporter in the distal convoluted tubule

PLOS ONE

Dear Dr. Kopp,

Thank you for submitting your manuscript to PLOS ONE. After careful consideration, we feel that it has merit but does not fully meet PLOS ONE’s publication criteria as it currently stands. Therefore, we invite you to submit a revised version of the manuscript that addresses the points raised during the review process.

Your manuscript was reviewed by two experts. Although they found your work interesting, they raised some concerns. Please revise it according to their suggestions. In addition, please consider performing genome-editing analyses as written in the "Additional Editor Comments" section below.

Please submit your revised manuscript by Jul 14 2022 11:59PM. If you will need more time than this to complete your revisions, please reply to this message or contact the journal office at [plosone@plos.org](mailto:plosone@plos.org). When you're ready to submit your revision, log on to <https://www.editorialmanager.com/pone/> and select the 'Submissions Needing Revision' folder to locate your manuscript file.

Please include the following items when submitting your revised manuscript:

- A rebuttal letter that responds to each point raised by the academic editor and reviewer(s). You should upload this letter as a separate file labeled 'Response to Reviewers'.
- A marked-up copy of your manuscript that highlights changes made to the original version. You should upload this as a separate file labeled 'Revised Manuscript with Track Changes'.
- An unmarked version of your revised paper without tracked changes. You should upload this as a separate file labeled 'Manuscript'.

If you would like to make changes to your financial disclosure, please include your updated statement in your cover letter. Guidelines for resubmitting your figure files are available below the reviewer comments at the end of this letter.

If applicable, we recommend that you deposit your laboratory protocols in protocols.io to enhance the reproducibility of your results. Protocols.io assigns your protocol its own identifier (DOI) so that it can be cited independently in the future. For instructions see: [https://journals.plos.org/plosone/s/submission-guidelines#loc-laboratory-protocols](https://gcc02.safelinks.protection.outlook.com/?url=https%3A%2F%2Fjournals.plos.org%2Fplosone%2Fs%2Fsubmission-guidelines%23loc-laboratory-protocols&data=05%7C01%7Cjeffreyk%40intra.niddk.nih.gov%7C8d2e7443f62e43ac9b8c08da423b1447%7C14b77578977342d58507251ca2dc2b06%7C0%7C0%7C637895118908202727%7CUnknown%7CTWFpbGZsb3d8eyJWIjoiMC4wLjAwMDAiLCJQIjoiV2luMzIiLCJBTiI6Ik1haWwiLCJXVCI6Mn0%3D%7C3000%7C%7C%7C&sdata=lwYW3t2549h9GHZ9sfJu0YqkuqJPghgPTmujlimNzAQ%3D&reserved=0). Additionally, PLOS ONE offers an option for publishing peer-reviewed Lab Protocol articles, which describe protocols hosted on protocols.io. Read more information on sharing protocols at [https://plos.org/protocols?utm_medium=editorial-email&utm_source=authorletters&utm_campaign=protocols](https://gcc02.safelinks.protection.outlook.com/?url=https%3A%2F%2Fplos.org%2Fprotocols%3Futm_medium%3Deditorial-email%26utm_source%3Dauthorletters%26utm_campaign%3Dprotocols&data=05%7C01%7Cjeffreyk%40intra.niddk.nih.gov%7C8d2e7443f62e43ac9b8c08da423b1447%7C14b77578977342d58507251ca2dc2b06%7C0%7C0%7C637895118908202727%7CUnknown%7CTWFpbGZsb3d8eyJWIjoiMC4wLjAwMDAiLCJQIjoiV2luMzIiLCJBTiI6Ik1haWwiLCJXVCI6Mn0%3D%7C3000%7C%7C%7C&sdata=azPeQbTqrxCJwQt3c7bQOnOFOYOpRFb94WoK2mYX0Yk%3D&reserved=0).

We look forward to receiving your revised manuscript.

Kind regards,

Hodaka Fujii, M.D., Ph.D.
Academic Editor
PLOS ONE

Journal Requirements:

When submitting your revision, we need you to address these additional requirements.

1.Please ensure that your manuscript meets PLOS ONE's style requirements, including those for file naming. The PLOS ONE style templates can be found at 
<https://journals.plos.org/plosone/s/file?id=wjVg/PLOSOne_formatting_sample_main_body.pdf> and 
<https://journals.plos.org/plosone/s/file?id=ba62/PLOSOne_formatting_sample_title_authors_affiliations.pdf>

**Author response:** We have formatted our manuscript as per instructions.

2. To comply with PLOS ONE submissions requirements, in your Methods section, please provide additional information regarding the experiments involving animals and ensure you have included details on (1) methods of sacrifice, (2) methods of anesthesia and/or analgesia, and (3) efforts to alleviate suffering.

**Author response:** We have included the requested information in the Materials and Methods section, Page 6 of manuscript.

3. We note that the grant information you provided in the ‘Funding Information’ and ‘Financial Disclosure’ sections do not match.

When you resubmit, please ensure that you provide the correct grant numbers for the awards you received for your study in the ‘Funding Information’ section.

**Author response:** We have provided the correct funding information including the grant number in the cover letter.

4.Thank you for stating the following in the Acknowledgments Section of your manuscript:

"This work was funded by the NIDDK Intramural Research Program. We appreciate assistance from Jurgen Heymann, Kris Ylaya, Ryan G Morris and Abeer Fadda."

We note that you have provided funding information that is not currently declared in your Funding Statement. However, funding information should not appear in the Acknowledgments section or other areas of your manuscript. We will only publish funding information present in the Funding Statement section of the online submission form. 
Please remove any funding-related text from the manuscript and let us know how you would like to update your Funding Statement. Currently, your Funding Statement reads as follows:

"This work was funded by the NIDDK Intramural Research Program."

**Author response:** We have deleted the funding information from the Acknowledgments Section. The funding information is now provided in the cover letter.

Please include your amended statements within your cover letter; we will change the online submission form on your behalf.

**Author response:** We have accordingly amended the statement in our cover letter.

5. Thank you for stating the following in the Competing Interests section:

"Author JBK holds a patent relating to monoclonal antibodies to HIV-1 Vpr and methods of using same.   United States Patent 7,993,647 (2015). No other conflicts of interest, financial or otherwise, are declared by the authors."

Please confirm that this does not alter your adherence to all PLOS ONE policies on sharing data and materials, by including the following statement: "This does not alter our adherence to PLOS ONE policies on sharing data and materials.” (as detailed online in our guide for authors <http://journals.plos.org/plosone/s/competing-interests>).  If there are restrictions on sharing of data and/or materials, please state these. Please note that we cannot proceed with consideration of your article until this information has been declared.

**Author response:** We have added the statement: "This does not alter our adherence to PLOS ONE policies on sharing data and materials.” in the “[Disclosure](http://www.icmje.org/disclosure-of-interest/) statement” section of the revised manuscript.

Please include your updated Competing Interests statement in your cover letter; we will change the online submission form on your behalf.

**Author response:** We have accordingly updated the statement in our cover letter.

6. PLOS ONE now requires that submissions reporting blots or gels include original, uncropped blot/gel image data as a supplement or in a public repository. This is in addition to complying with our image preparation guidelines described at <https://journals.plos.org/plosone/s/figures#loc-blot-and-gel-reporting-requirements>. These requirements apply both to the main figures and to cropped blot/gel images included in Supporting Information. If the manuscript is positively reviewed, we will ask the authors to provide any missing raw image data for blot/gel results when they submit their first revision. As part of your review, please ensure that figures reporting blot or gel images comply with the journal’s image preparation guidelines and that the original data are provided following the journal’s request.  If you have any questions or concerns about blot/gel figures or data for this submission, please email us at [plosone@plos.org](mailto:plosone@plos.org) before issuing a decision letter.

**Author response:**  Supplement document contains all uncropped blot/gel of Fig. 3 (pages 13, 14 , Supporting Information) and S4 Fig (page 14, Supporting Information). The tif files of figures in main text have been submitted separately.

7. We note that you have included the phrase “data not shown” in your manuscript. Unfortunately, this does not meet our data sharing requirements. PLOS does not permit references to inaccessible data. We require that authors provide all relevant data within the paper, Supporting Information files, or in an acceptable, public repository. Please add a citation to support this phrase or upload the data that corresponds with these findings to a stable repository (such as Figshare or Dryad) and provide and URLs, DOIs, or accession numbers that may be used to access these data. Or, if the data are not a core part of the research being presented in your study, we ask that you remove the phrase that refers to these data.

**Author response:** In compliance with the PLOS publication policy, we have withdrawn the phrase “data not shown” from page 23 (original version) of the Discussion section, because the data are not a core part of the research being presented and deleting these lines does not interfere with major findings and the interpretations of this study.

Additional Editor Comments:

It would be preferable to mutagenize MRE and SP1 motifs in the endogenous gene promoter using genome editing techniques and examine expression levels of the SLC12A3 transcript.

**Author response:** : While we agree with the editor’s suggestion to engineer MRE and SP1 mutations by CRISPR/Cas or other gene editing techniques and characterize the resulting mice, however, this endeavor will take at least one year. We feel that the resubmitted manuscript provides convincing data supporting a role for Vpr in suppressing *SLC12A3/Slc12a3* gene transcript by interfering with the binding of MR to the MRE of *SLC12A3/Slc12a3* gene promoter region and decrease in NCC protein abundance. This subsequently results in HIV-associated salt wasting, shown in an *in vivo* model (Vpr transgenic mice). The mechanisms related to the decrease in NCC abundance is shownin an *in vitro* model (*SLC12A3* promoter elements in human distal convoluted tubule cells and African green monkey kidney (CV-1) cells, Fig 6).

[Note: HTML markup is below. Please do not edit.]

Reviewers' comments:

Reviewer's Responses to Questions

**Comments to the Author**

1. Is the manuscript technically sound, and do the data support the conclusions?

The manuscript must describe a technically sound piece of scientific research with data that supports the conclusions. Experiments must have been conducted rigorously, with appropriate controls, replication, and sample sizes. The conclusions must be drawn appropriately based on the data presented.

Reviewer #1: Yes

Reviewer #2: Yes

2. Has the statistical analysis been performed appropriately and rigorously?

Reviewer #1: Yes

Reviewer #2: Yes

3. Have the authors made all data underlying the findings in their manuscript fully available?

The [PLOS Data policy](https://gcc02.safelinks.protection.outlook.com/?url=http%3A%2F%2Fwww.plosone.org%2Fstatic%2Fpolicies.action%23sharing&data=05%7C01%7Cjeffreyk%40intra.niddk.nih.gov%7C8d2e7443f62e43ac9b8c08da423b1447%7C14b77578977342d58507251ca2dc2b06%7C0%7C0%7C637895118908202727%7CUnknown%7CTWFpbGZsb3d8eyJWIjoiMC4wLjAwMDAiLCJQIjoiV2luMzIiLCJBTiI6Ik1haWwiLCJXVCI6Mn0%3D%7C3000%7C%7C%7C&sdata=yO2QYHcGItD%2FuCdlpU8PTV4mDkQRS3Mvh053MK6gRhM%3D&reserved=0) requires authors to make all data underlying the findings described in their manuscript fully available without restriction, with rare exception (please refer to the Data Availability Statement in the manuscript PDF file). The data should be provided as part of the manuscript or its supporting information, or deposited to a public repository. For example, in addition to summary statistics, the data points behind means, medians and variance measures should be available. If there are restrictions on publicly sharing data—e.g. participant privacy or use of data from a third party—those must be specified.

Reviewer #1: Yes

Reviewer #2: Yes

4. Is the manuscript presented in an intelligible fashion and written in standard English?

PLOS ONE does not copyedit accepted manuscripts, so the language in submitted articles must be clear, correct, and unambiguous. Any typographical or grammatical errors should be corrected at revision, so please note any specific errors here.

Reviewer #1: Yes

Reviewer #2: Yes

5. Review Comments to the Author

Please use the space provided to explain your answers to the questions above. You may also include additional comments for the author, including concerns about dual publication, research ethics, or publication ethics. (Please upload your review as an attachment if it exceeds 20,000 characters)

Reviewer #1: Vpr-induced salt wasting and loss of NCC protein expression in mouse kidney was well studied here and, in parallel, Vpr-induced decrease in genomic bindings of MR to Slc12a3 gene location and that in the aldosterone-dependent transcriptional activity were also well demonstrated in renal cell-lines**.** However, considering the difficulty in detecting a change of MR genomic bindings in in-vivo DCT, the author’s hypothesis would have to be more supported by other data which associates these two parts of the study closely.

**Author response to Reviewer 1 main critique**: We tested the hypothesis that Vpr acts as a repressor of MR activity and thereby promotes suppression of aldosterone-stimulated NCC gene expression in the distal nephron, contributing to renal salt wasting. The Vpr-mediated suppression of MR binding to MRE of *Slc12ac* results in the suppression of *Slc12ac* transcription and reduction of NCC protein expression and salt wasting. We agree with the reviewer that a weakness of the manuscript is the lack of a direct connection between the MRE decificeny (or blocking binding of MR with MRE) and the reduction in NCC protein expression *in vivo*. As stated in our response to the editor, it would be ideal to engineer MRE and SP1 mutations by CRISPR/Cas in human distal convoluted tubule (hDCT) cells and mice, and characterize the resulting hDCT cells and mice. However, it will take considerable effort to do so. We hope the reviewer and editor will agree that the current data are sufficient to support a role of Vpr in suppressing MR binding to MRE of *Slc12a3*, which results in the suppression of its transcription and reduction of NCC abundance and subsequent salt wasting.

Main concerns:
1. Not only the genomic bindings of MR by Vpr treatment in human DCT cell-line, was the endogenous expression level of Slc12a3 also decreased? At least, such changes in expression level of NCC protein and/or mRNA should be demonstrated.

**Author response to reviewer #1 main concern (1) :** We agree with the reviewer’s comment, and to obviate this concern, we have included the requested data (new **S3 Fig**). Using hDCT cells we quanified the mRNA (qRT-PCR) and protein (western blot) expressions of *SLC12A3*/NCC. This figure (new **S3 Fig**) shows the downregulation of endogenous expression of NCC in hDCT cells at both mRNA and protein levels, which is consistent with our *in vivo* observations (Figures 3A and 3C; Figures 5A and 5B).

2. As shown in Fig 3a, NCC protein level was clearly diminished in the kidney of Vpr-transgenic mice compared to that of wild-type mice. However, the intensity of signal in each of positive-cells in IHC and in-situ hybridization as shown in Fig. 3c and 5b seems strong almost equally in both of the groups of mice, leading to a doubt that the Vpr-induced change in renal NCC protein level would not be derived from the cellular expression level of Slc12a3 but the number of DCT cells in the kidney. At least, it should be shown that the number of DCT cells in each of the sections were not affected with the transgenic expression of Vpr.

**Author response to reviewer #1 main concern (2):** The reviewer’s comment regarding Fig3C and Fig 5B are well taken, and we agree that the similar intensity of *Slc12a3* signal in individual positive cells in fig 5B, could be caused by both the loss or reduction in mRNA. There are less mRNA-positive cells in kidney of Tg than WT mice, especially after salt depletion. The possibility of DCT cell loss in HIV-1 Vpr transgenic mice, is addressed in page 20, Discussion section. This topic is under investigation and will be submitted as part of another manuscript.

We hope that the reviewer will accept the current manuscript for publication as it provides adequate evidence that the HIV-1 Vpr suppreses MR binding to the MREs of *Slc12a3* promoter regions, the extent of which may be uneven from cell to cell.

3. About the supplemental Fig 3, the staining pattern of NCC seems rather cytoplasmic, which does not indicate strong expression of NCC in plasma membrane. More conservative statement would be more acceptable for readers.

**Author response:** We agree with the reviewer that it is difficult to distinguish between NCC expression at the plasma membrane and in cytoplasm in supplementary Fig. 4 (Supplementary figure 3 in the original version). As suggested by the reviewer, we have modified the figure legend accordingly by deleting “plasma membrane”~~.~~ We noted that similar pattern of the NCC staining has been noted in previous reports using the same antibody as ours Zhou et al, JASN, 2013; Louis-Dit-Picard et al, Nat Genet. 2012). The endogenous NCC intracellular distribution is similar to the exogenous GFP-NCC expressed in both plasma membrane and cytoplamsa (Zhou et al, JASN, 2010).

Minor concern:
1. Some typos are observed, such as doubling of “PMC” in the reference section.

**Author response:** We have corrected this and other typos throughout the manuscript.

Reviewer #2: The work is very well done, experiments are well controlled and supporting evidence from various sources is nicely used. The work clearly supports an effect of VPR in the renal tubules, specifically upon MR and then NCC. My main critique of the manuscript is that this observed effect upon NCC may not be the entirety or even the major mechanism by which HIV/VPR mediated sodium wasting occurs.

**Author response to main critique:** The current manuscript describes that Vpr suppresses the binding of MR to the MRE of *Slc12a3*, which results in the reduction of its transcription and subsequent NCC protein expression. We agree with the reviewer that 1) in addition to NCC, other sodium transporters/channels/pumps along nephron segments could contribute to Vpr-mediated sodium wasting as well; 2) in addition to MRE other mechanism (s), such as atrophy of DCT cells, could also contribute to the Vpr-medicated reduction of NCC abundance (discussed in the last paragraph of page 20), which needs further investigation (also mentioned in our responses to the editor and Reviewer 1).

Questions: **Reviewer #2 question was split in to questions A, B, and C.**

A) Why aren’t aldo and renin levels increased compared to controls?
   B) Mice were fed the same chow, but was intake controlled or did actual intake vary among the mice (I expect so but not explicitly stated)? In a study of Gitelman’s patients, Lifton famously found that sodium intake among affected subjects (and heterozygotes) increased compared to unaffected individuals. C)Is it possible that the low sodium chow designed to induce increased aldo levels prevented us from seeing a further physiologic rise in renin/aldo? Does this in turn prevent the usual compensatory increases in sodium reabsorption along the nephron (ENaC specifically)?

Question a: Why aren’t aldo and renin levels increased compared to controls?

**Authors responses to Question A**:

The plasma renin activity and plasma aldosterone levels were increased to a similar degree with chronic salt depletion in the Tg and WT groups. The 24-hour sodium excretion was 45.74% greater in the Tg than WT group before chronic salt depletion and was decreased by chronic salt depletion to a similar exent in the Tg group (-22.62%) and WT group (-25.09%) (calculated from **Fig 2A**). However, urine flows were similar in the two groups before and after salt depletion; urine flow was not decreased by chronic salt depletion (**S3 Fig**). The body weights were also similar in the Tg and WT groups with chronic salt depletion (**S3 Fig**). It is possible that compensatory mechanisms related to maintenance of fluid balance that remain to be discovered prevented a greater increase in the plasma renin activity and plasma aldosterone levels in the Tg group that the WT group. The effect on plasma renin activity is discussed in the first paragraph, Page 18). The role of aldosterone is discussed in page 18, second and third paragraphs).

As the reviewer knows, major regulators of aldosterone secretion are K^+^ and Ang II (among others, e.g., Ang III, ACTH, adrenoglomerulotropin, etc). In our experiments plasma concentration of K^+^ was similar in WT and Tg mice (**S2 B Fig**). We did not measure serum/plasma Ang II levels in these mice.

Based on motif analysis, we did not identify genes of REN, AGT, and ACE as the MR or GR targeting genes. Most likely, Vpr does not bind to the promoter regions of these genes, and therefore Vpr most likely would not affect the protein expression of renin and Ang II production (expression of renin, angiotensinogen, and angiotensin I converting enzyme affects Ang II production) through MR/GR activity, which is consistent with a recent study that examined Vpr-targeted genes (Greenwood et al, Cell Reports 2019).

Our observation is also consistent with a recent clinical study that showed that low sodium diet decreased urinary sodium excretion to a similar degree in non-HIV and HIV; low sodium diet activated the renin-angiotensin-aldosterone system to a similar degree in both groups (Srinivasa et al, J Infect Dis, 2016).

Question b: Mice were fed the same chow, but was intake controlled or did actual intake vary among the mice (I expect so but not explicitly stated)? In a study of Gitelman’s patients, Lifton famously found that sodium intake among affected subjects (and heterozygotes) increased compared to unaffected individuals.

**Author response to Question B:** The reviewer is correct that Lifton et al reported increased salt intake in Gitelman patients, most possibly to compensate for the urinary sodium loss and maintain a normal plasma sodium level. As stated in response to Question A, the 24-hour sodium excretion was 45.74% greater in the Tg than WT group before chronic salt depletion and was decreased by chronic salt depletion to a similar exent in the Tg group (-22.62%) and WT group (-25.09%) (calculated from **Fig 2A**). However, urine flows were similar in the two groups before and after salt depletion (**S3 Fig**); urine flow was not decreased by chronic salt depletion. The body weights were also similar in the Tg and WT groups with chronic salt depletion (**S3 Fig**).

It is possible that prior to the four-day salt depletion period, the Tg mice consumed more food/salt to maintain a normal sodium balance. During the four-day salt-depletion period, both WT and Tg mice were fed the same measured amount of food daily. Therefore, we assume that the mice had consumed the same amount of sodium with ad libitum access to drinking water. Our failure to record the food/salt intake prior to the four day salt depletion is a limitation, that we have acknowledged in Discussion section (page 21, first paragraph). We also cited the relevant Lifton reports (References 58 and 59).

Question c: Is it possible that the low sodium chow designed to induce increased aldo levels prevented us from seeing a further physiologic rise in renin/aldo? Does this in turn prevent the usual compensatory increases in sodium reabsorption along the nephron (ENaC specifically)?

**Author response to Question C:** The reviewer is correct that the low sodium chow was designed to stimulate aldosterone secretion, similar to the *in vitro* experiments in which exogenous aldosterone was added in culture media of the hDCT and CV-1 cells to activate MR. However, we do not think that the increased aldosterone induced by the low salt diet prevented a further increase in renin/aldosterone in Vpr Tg mice, since there was no greater increase before salt depletion in Tg mice compared to WT mice. Nevertheless, we agree that in the context of salt wasting described in this report, the role of ENaC needs further investigation.

I agree with the investigators that “Whether or not transgenic expression alters the aldosterone/MR-mediated gene and/or protein expression of sodium transporters and exchangers (e.g. Na+-K+-2Cl- cotransporter/NKCC2 and epithelial sodium channels/ENaCs) along the nephron segment requires further investigation.” While the component of NCC in this phenomenon could be examined with a series of laborious thiazide treatments, this is likely to be of limited scientific value to the human disease studied due to differences in tubule contributions to sodium reabsorption across species. I would be sure to caveat my conclusions to indicate that possibility that NCC only contributes to this effect.

**Author response:** As discussed above, we agree with the reviewer that, in addition to NCC, other sodium transporters/channels/pump should be investigated in future studies.

6. PLOS authors have the option to publish the peer review history of their article ([what does this mean?](https://gcc02.safelinks.protection.outlook.com/?url=https%3A%2F%2Fjournals.plos.org%2Fplosone%2Fs%2Feditorial-and-peer-review-process%23loc-peer-review-history&data=05%7C01%7Cjeffreyk%40intra.niddk.nih.gov%7C8d2e7443f62e43ac9b8c08da423b1447%7C14b77578977342d58507251ca2dc2b06%7C0%7C0%7C637895118908202727%7CUnknown%7CTWFpbGZsb3d8eyJWIjoiMC4wLjAwMDAiLCJQIjoiV2luMzIiLCJBTiI6Ik1haWwiLCJXVCI6Mn0%3D%7C3000%7C%7C%7C&sdata=2vz5zCVjZ2G2cMI1GT%2BhB7N45Sj5TBJWYEYo34fKiFU%3D&reserved=0)). If published, this will include your full peer review and any attached files.


If you choose “no”, your identity will remain anonymous but your review may still be made public.


**Do you want your identity to be public for this peer review?** For information about this choice, including consent withdrawal, please see our [Privacy Policy](https://gcc02.safelinks.protection.outlook.com/?url=https%3A%2F%2Fwww.plos.org%2Fprivacy-policy&data=05%7C01%7Cjeffreyk%40intra.niddk.nih.gov%7C8d2e7443f62e43ac9b8c08da423b1447%7C14b77578977342d58507251ca2dc2b06%7C0%7C0%7C637895118908202727%7CUnknown%7CTWFpbGZsb3d8eyJWIjoiMC4wLjAwMDAiLCJQIjoiV2luMzIiLCJBTiI6Ik1haWwiLCJXVCI6Mn0%3D%7C3000%7C%7C%7C&sdata=Ht5XdR8d89B%2BYLpkg6hHfuFxDQXN%2B80GrzpOyuBK6Bc%3D&reserved=0).

Reviewer #1: **Yes:** Ueda Kohei

Reviewer #2: No
